# Annual climatic fluctuations and short-term genetic variation in the eastern spadefoot toad

**DOI:** 10.1038/s41598-021-92696-w

**Published:** 2021-06-29

**Authors:** Orly Cohen, Yoav Ram, Lilach Hadany, Sarig Gafny, Eli Geffen

**Affiliations:** 1 School of Zoology, Tel Aviv University, 69978 Tel Aviv, Israel; 2 School of Plant Sciences and Food Security, Tel Aviv University, 69978 Tel Aviv, Israel; 3 Faculty of Marine Sciences, Ruppin Academic Center, 40297 Michmoret, Israel

**Keywords:** Ecological genetics, Evolutionary ecology, Ecology, Evolution, Genetics, Zoology

## Abstract

In addition to variations on the spatial scale, short- and long-term temporal variations, too, can impose intense selection on the overall genetic diversity and composition of a population. We hypothesized that the allelic composition in populations of the eastern spadefoot toad (*Pelobates syriacu*s) would change among successive years in accordance with the short-term changes in environmental conditions. Surprisingly, the effect of short-term climate fluctuations on genetic composition have rarely been addressed in the literature, and to our knowledge the effect of annual climatic fluctuations have not been considered meaningful. Our findings show that climatic variation among successive years, primarily the amount of rainfall and rainy days, can significantly alter both microsatellite allelic composition and diversity. We suggest that environmental (i.e. fluctuating) selection is differential across the globe, and that its intensity is expected to be greatest in regions where short-term climatic conditions are least stable.

## Introduction

Most population genetic studies have focused on the spatial scale, based on samples collected at a single point in time and on the assumption that the observed genetic structure remains stable over short-term periods. When temporal genetic diversity is addressed, it has been mostly tested on long-term periods spread over decades, using preserved historical samples from museums (e.g.,^1–4^). There are only a few studies that have examined the changes in genetic characteristics over short-term periods; and most of these were conducted on aquatic species of economic importance in order to determine genetic stability under harvesting pressure (e.g.,^5–8^). Repeated sampling over short-term periods can reveal correlations between demographic and genetic changes^9,10^; can help to detect higher genetic variation and rare genotypes that mostly appear in a single year^11^; and contribute to monitoring the progress of conservation management practices^12^. Consequently, there is growing attention to the study of short-term temporal genetic variation in populations of wild animals.

Amphibians present good models by which to examine theories regarding short-term temporal genetic variations in populations of wild animals because they are widely distributed in most ecosystems, easy to sample in breeding assemblages, and often philopatric to breeding sites^13–16^.

In the present work, we extended the study by Munwes et al.^17^ to focus on the influence of short-term climatic processes on genetic variation in the eastern spadefoot toad (*Pelobates syriacus*) in Israel. The eastern spadefoot toad is a fossorial species, spending the hot and dry periods in burrows in the soil. Its spawning sites comprise stagnant temporary water bodies, rivers or lakes, and large permanent pools^18^. In Israel, this species is known to breed only in ephemeral pools and the adults are philopatric, breeding in the same vernal pool in consecutive years^19^. The distribution range of *P. syriacus* extends to southern Romania in the north, Greece in the west, Iran in the east, and Israel in the south^18^. In Israel, it occurs from the Golan Heights and Galilee in the north, through the central coastal plain, which includes its present southernmost breeding site—Robert's pool near Ashdod^17^. Spadefoot toad species display extreme variation in larval period duration, due in part to evolution of thyroid hormone (TH) physiology^20^. Eastern spadefoot toad tadpoles exhibit plasticity in the timing of metamorphosis^21^, and environmental factors have been shown to affect different traits in the tadpoles, allowing rapid development in drying ephemeral ponds^22^. At each pool spawning is usually happens within two to three weeks, during nights of peak heavy rain^17,19^. Metamorphosis at each rainpool is a synchronous event; the tadpoles metamorph within a period of 1 week^19^. In the coastal plains, on years with a short hydroperiod, tadpoles can already metamorph after 2.5 months. On years with a long hydroperiod, metamorphosis was observed only at 5–6 months. In the northern pools, where hydroperiods are even longer, tadpoles can stay in the water up to 8 months^17,19^. Metamorphs were observed up to 1 km away from their natal rainpool shortly after leaving the water. Adults are philopatric, breeding in the same rainpool in consecutive years, and were not observed further than 200 m from the water edge^17,19^. From a capture-recapture study in several rainpools, the estimated population size for each pool ranged 22–26 breeding adults^19^. Out of 50 adults that were collected from two pools in 1983–85, only three individuals were of age 2 years or less.

**Figure 1:**
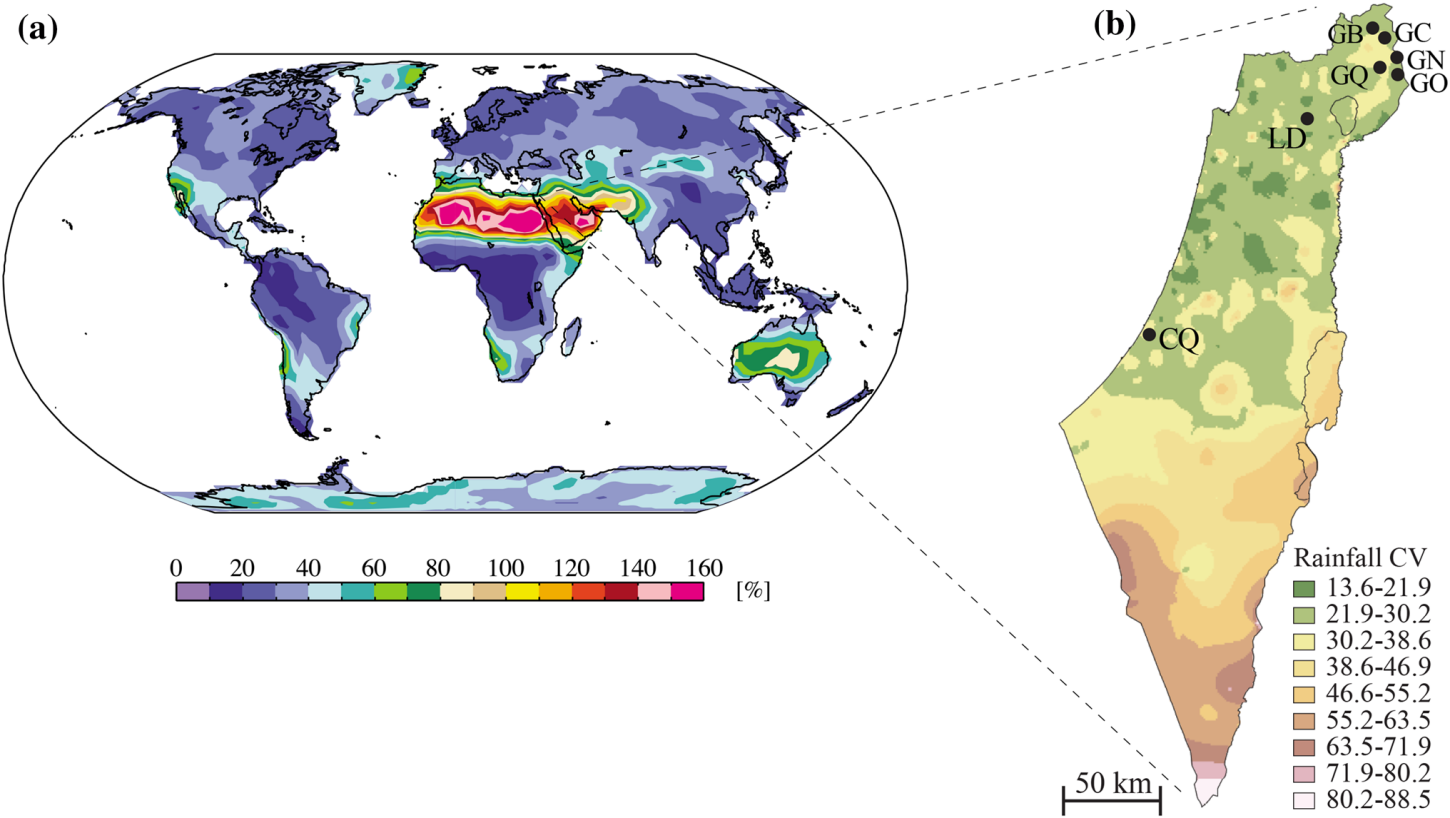
(**a**) Model for the global interannual variability in rainfall (CV; modified after Mahlstein et al.^23^). All results are for the wet season. (**b**) Map of Israel denoting the seven vernal pools sampled in the study. The color patterns show the coefficient of variation (CV, %) in annual rainfall between the years 1999–2018. Rainfall data was obtained from the Israel Meteorological Service (IMS), and the annual rainfall variation layer was constructed using ArcGIS (version 10, ESRI Inc.). Pool name abbreviations were taken from Table S2.

**Figure 2:**
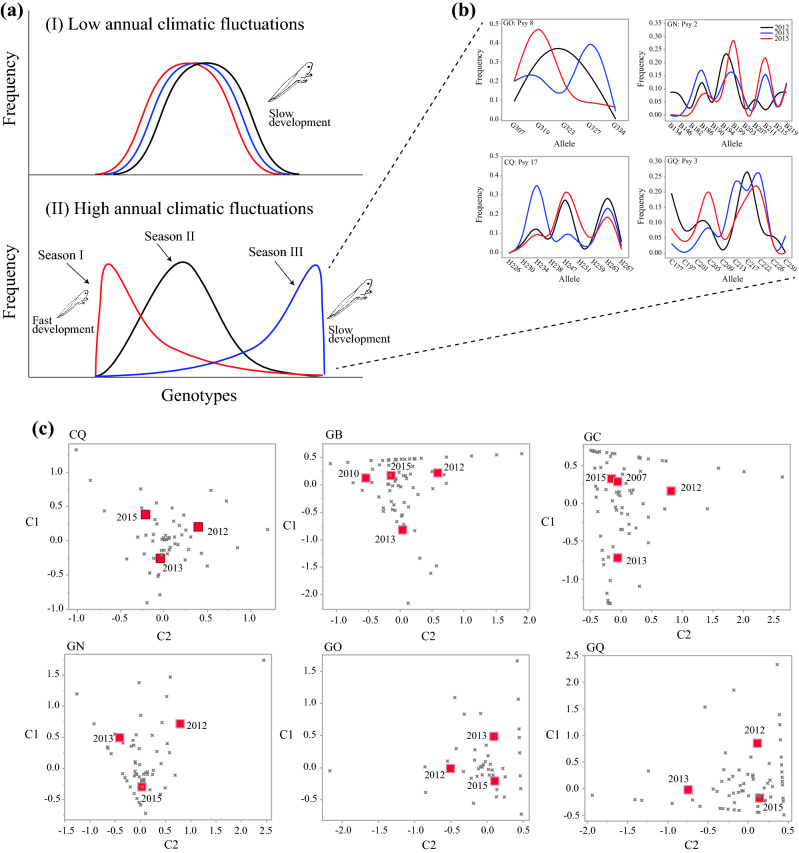
(**a**) The hypothesized frequency distribution of alleles in successive years in pools located in areas of low annual climatic fluctuations (**a.I**) and high annual climatic fluctuations (**a.II**). As an example of the variation in selection among successive years, the large tadpole image is associated with a long hydroperiod and slow growth rate, while the small tadpole image is associated with a short hydroperiod and fast growth rate. See text for more details. (**b**) The interannual variation in allele distribution for four loci (Psy 2, 3, 8, 17) and vernal pools (CQ, GN, GO, GQ) is provided as an example of the high annual fluctuations in the study area. (**c**) Correspondence analysis associating allele frequencies (gray crosses) and years (red squares) in six vernal pools. Pool name abbreviations were taken from Table S2.

Israel is situated in a zone where interannual variation is high (Fig. 1a,^23^). We hypothesized that the genetic make-up of the tadpole population (i.e. diversity and frequencies of alleles) would change among successive years in accordance with changes in environmental conditions (Fig. 2a). Climate and environmental conditions (e.g., temperature, hydroperiod, etc.) may affect the intensity and trajectory of selection in a given year, giving rise to differences in genetic variation among years. For example, let us assume that some tadpoles are also genetically inclined to slow development, while others are also genetically inclined to fast development. Fast development is advantageous in dry years, when the hydroperiod of vernal pools is short. Tadpoles that develop fast will reach metamorphosis before the pool is dry, and thus survive (Fig. 2a.II). In contrast, slow development is advantageous in years with heavy rainfall, when the hydroperiod is lengthy. Tadpoles with the ability for slow development will be able to grow larger, store more resources, and spend less time in aestivation on land than those that have metamorphosed earlier. Thus, slow development provides an increased survival in years of heavy rainfall (Fig. 2a.II). The above scenario is just a simplified example to illustrate how short-term changes in environmental conditions can affect a variety of traits (e.g. tolerance of high salinity, high levels of H_2_S, and increased hypoxia). Consequently, in habitats in which environmental conditions vary significantly from year to year each generational cohort can potentially possess a different composition of genotypes or alleles (Fig. 2a.II). Under a more predictable climatic regime, when living and breeding conditions remain generally similar from year to year, the selection pressure displays a similar intensity and trajectory, and, therefore, a more similar distribution of genotypes/alleles will survive to adulthood each year (Fig. 2a.I). Combining samples from a given population, irrespective of individual age (i.e. individuals hatched in different years), is a common practice in population genetics studies. Combining samples from different years could hypothetically result in higher genetic diversity at sites with a temporally fluctuating selection regime, compared with sites experiencing more stable temporal conditions^17^. This hypothesis is related to the concept that associates genetic variation at edge and core populations to climatic stability^24–27^.

Following the above hypothesis (Fig. 2a), we predicted that interannual climatic fluctuations would affect the interannual variation in genetic diversity and allelic composition of *P. syriacus* in any given vernal pool we examine. Through a series of tadpole sampling events from each pool over several consecutive years, and genetic analyses using eight microsatellite markers, we tested the above hypothesis and prediction. Our results are expected to provide a greater understanding of the processes that shape genetic structure and variation in populations subjected to high annual fluctuations in climate, and on the possible longer-term effects of climate change on population genetics.

## Methods

### Sample collection

The study was conducted in seven vernal pools known as breeding sites for *Pelobates syriacus* in Israel (Fig. 1). The sampling sites were from three geographical regions: Golan Heights (GH), Lower Galilee (LG), and the Coastal Plain (CP), which includes the most southern known breeding site for this species (Fig. 1b). DNA samples were collected over a period of four years (2012–2015). We also included additional DNA samples collected from 2006 to 2010 by Munwes et al.^17^.

We visited each vernal pool 2–3 times a year, during winter and spring (Oct–May), and each time sampled 15–20 tadpoles. Most pools are small (diameter < 50 m) and shallow (depth < 1 m), enabling extensive sampling. No metamorphs were observed during any of our sampling sessions; thus, all our sampling has been done before any tadpoles metamorphed and left the pool. To maximize the chance of sampling tadpoles from different spawns (i.e. individuals that are not full siblings), we intensively sampled from around each pool. Captured tadpoles were measured (snout to tail tip). Newly-hatched tadpoles (length ≤ 3 cm) were collected whole and tail-tip samples were taken from larger tadpoles. All samples were stored in 96% alcohol and kept frozen until analysis.

### Microsatellite analyses

We developed novel microsatellite loci (Table S1), a set of genetic markers that is particularly suitable for individual-level analysis^28^. Genomic DNA samples from *P. syriacus* were sent to the Evolutionary Genetics Core Facility (EGCF) at Cornell University for genomic library construction via Illumina MiSeq sequencing. The EGCF provided an msatcommander^29^ output file with thousands of potential microsatellite primer pairs. Further details on the selection process of microsatellites for this analysis are provided in Table S1.

### Climate and environmental information

According to our hypothesis, we expected to find significant changes in allele composition and diversity between successive years due to changes in the trajectory and intensity of environmental selection. In addition to climate, there are other environmental factors that may affect tadpole survival and thus also genetic variability. Munwes et al.^17^ investigated the effect of elevation, distance to the Mediterranean Sea, vegetation type, and soil type on genetic diversity. They found that a combination of hydroperiod, annual rain, and altitude; or a combination of hydroperiod, soil type, and vegetation, best explained the variance in mtDNA among sites (Fst) and accounted for about 17% of the variance. These variables are relevant when testing for spatial genetic patterns. However, when testing for short-term temporal patterns, variables such as elevation, soil type, and vegetation cover do not change much from year to year. Consequently, we did not to include these variables in the analysis. We chose to focus on four annual environmental variables of high relevance for spadefoot toad breeding behavior and tadpole survival: the mean daily maximum temperature in January (MJT), the annual rainfall (AR), the number of days with more than 0.1 mm rain (RD), and the hydroperiod length (HP). We selected to use both hydroperiod and rainfall predictors because in our study system heavy rainfall occasionally occurs late in the season (April–May, 1–2 rainy days). Such short but intense rainstorms can refill pools that were nearly dry, and in which most of the tadpoles have already metamorphed, and extend the hydroperiod by a few more weeks. Thus, rainfall and days of rain may contain more relevant information on tadpole survival compared to hydroperiod duration alone. Climate variables were obtained from the Israel Meteorological Service (IMS) database (https://ims.data.gov.il/), in which the annual rainfall and number of rainy days are measured between August 1st of one year and July 31th of the next year. Accordingly, in this study, we refer to a sampling season as the time between August of a given year and July the year after. The IMS has meteorological stations spread across the country, and the climate data were taken from the closest meteorological station to each vernal pool. In cases where there was no close station to the pool, we used the average of two or three stations in the area (Table S2). The length of the hydroperiod (HP) was evaluated according to the condition of the pool during the sampling sessions throughout the year, following the methodology of Gafny and Gasith^30^. The correlation between all four environmental variables (Table S3) and the variance inflation factor (VIF) were used to evaluate the level of collinearity.

### Annual change in allelic diversity

We evaluated genetic diversity using three common measures: mean number of alleles per locus (Na), observed heterozygosity (Ho), and Shannon's information evenness (J = SI/SI_max_, SI = Shannon's information index^31^). These diversity measures were calculated per year and pool using GeneAlEx (version 6.5^32^). The presence of null allele, stuttering and large allele dropout was tested with Micro-Checker (version 2.2.3^33^). Deviation from Hardy–Weinberg equilibrium (HWE) and evidence of linkage disequilibrium (LD) were tested as implemented in Genepop (version 4.4^34^). Since the number of annual samples varied among vernal pools, we standardized Na values within each pool.

The effect of the climatic parameters on each of the genetic diversity measures was evaluated using a mixed model under the Generalized Estimating Equations (GEE) framework in SPSS (version 26; IBM). In these models, the four environmental parameters (MJT, AR, RD, HP) were set as fixed effects. In addition, the number of annual samples per pool was added as a covariate, and the vernal pool identity was set as the random effect. Finally, we standardized the climatic variables and Na values within each pool in order to control for geographical difference among pools.

### Annual change in allelic composition

To test for changes in allele composition among years we applied two approaches. First, we counted the numbers of alleles in each year for each pool, and constructed a contingency table. We used the Chi-Square test to test for significant differences in allele frequencies among years. Since many alleles had a low frequency (< 5), we used a randomization test (10,000 permutations) to avoid sample size bias. We used correspondence analysis to visualize the differences among years and to associate between alleles and year. Second, to evaluate the temporal component in genetic variation we calculated the corrected Nei’s average number of differences in allele frequency between years within each pool (D_A_^35^) using ARLEQUIN (version 3.5^36^). To test whether the pairwise D_A_ values (between years within pools) significantly differed from those expected by random, we used a randomization test and 10,000 permutations.

The temporal component of genetic variation was evaluated using the locus-by-locus AMOVA approach as implemented in the ARLEQUIN package. For this analysis, we partitioned the variance components into the variance among vernal pools (spatial component), among years within vernal pools (temporal component), among individuals within a pool (the same pool and year), and within individuals.

The correspondence analysis provided the distinction between years according to the allele frequencies. To assess which climate variables have a significant effect on allele frequencies, we used a GEE mixed model with gamma distribution and log as the link function. All four environmental parameters (MJT, AR, RD, HP) were standardized within each pool and were set as fixed effects, and the vernal pool identity was set as the random effect. We assessed variable importance (VI) using the Independent Resampled Inputs approach, which evaluates the contribution of each of the factors in a model by random sampling, independently of the model type and fitting method^37^).

### Ethics

This study was conducted with permits from the Israeli Nature and Parks Authority for capturing and sampling populations of the eastern spadefoot toad across Israel (2012/38376, 2013/39322, 2015/40845). All procedures performed in this study were in accordance with the ethical standards of the Israeli Nature and Parks Authority.

## Results

### Sample collection

During the years 2012–2015, we sampled DNA from seven vernal pools in Israel. Unfortunately, the winter of 2013–2014 was a drought year in Israel with only one significant rainfall event in mid-December 2013. Most of the pools in the coastal plain were completely dry by mid-January 2014, and the pools in the Golan and Galilee also dried up very early in that season, i.e. before the breeding peak. Therefore, no tadpoles were collected from any of those sites during that winter.

### Microsatellite markers

Using a set of novel microsatellite markers (Table S1), we successfully genotyped 540 samples of *P. syriacus* DNA from seven vernal pools. All eight loci showed high polymorphism, with allele numbers ranging from 19 to 36 (Table S1). The mean (± SE) observed heterozygosity across all populations and loci was 0.739 ± 0.015, and mean (± SE) expected heterozygosity was 0.727 ± 0.011. No significant heterozygote excess was detected for any loci (*P* ≥ 0.06) or in any population (*P* ≥ 0.82). Significant heterozygote deficit was detected for three loci (Psy2: *P* < 0.001, Psy6: *P* < 0.001, Psy8: *P* = 0.004), and in four pools (GB: *P* < 0.001, GC: *P* = 0.012, GO: *P* < 0.001, GQ: *P* < 0.001). No allele dropout or stuttering was detected at any locus. The possible presence of a null allele was detected only at locus Psy2.

### Annual variation in genetic diversity

The annual mean number of alleles per locus (Na), the Shannon's information evenness (J), and the observed heterozygosity (Ho) for each sampled pool varied substantially (Table S4). The mean pool coefficient of variation (CV ± SD) between years in the number of alleles was 18.61% ± 11.33. However, the coefficients of variation between years in Shannon's evenness (7.29% ± 4.68) and in observed heterozygosity (4.81% ± 2.16) were considerably lower.

We examined whether the annual variations in Na, J, and Ho could be explained by annual climatic and environmental fluctuations. The annual variations in Na, J, and Ho were associated with the climatic variables, either directly or through the interactions among them (Table 1). Shannon's evenness significantly decreased with the increase in annual maximum January temperature. In the shortest hydroperiod, Shannon's evenness significantly decreased with the increase in the annual number of rainy days, whereas in the longest hydroperiod it increased with the increase in the annual number of rainy days (Table 1).

**Table 1:** The effect of maximum January temperature (C), annual rainfall (mm), number of rainy days (> 0.1 mm/day), and length of hydroperiod (days) on genetic diversity measures (mean number of alleles/locus, Shannon's information evenness, and observed heterozygosity).

Diversity measure	Number of alleles/locus (Na)			Shannon's evenness (J)			Observed heterozygosity (Ho)		
	Estimate	Wald ${\upchi }_{1}^{2}$	*P*	Estimate	Wald ${\upchi }_{1}^{2}$	*P*	Estimate	Wald ${\upchi }_{1}^{2}$	*P*
Maximum January temperature (MJT)	0.07	0.04	0.848	− 0.28	10.29	**0.001**	− 0.060	9.53	**0.002**
Annual rainfall (AR)	− 0.40	7.82	**0.005**	0.11	1.27	0.259	0.010	0.13	0.719
Annual rain days (RD)	− 0.55	18.46	**< 0.001**	− 0.05	4.20	**0.041**	0.021	2.85	0.091
Hydroperiod (HP)	0.06	0.35	0.557	0.06	2.75	0.097	0.031	20.05	**< 0.001**
MJT * AR	− 0.83	7.21	**0.007**	0.19	1.90	0.168	0.056	2.96	0.085
MJT * RD	− 0.37	13.33	**< 0.001**	− 0.05	2.01	0.156	− 0.040	10.15	**0.001**
MJT * HP	0.05	0.02	0.890	− 0.03	0.41	0.523	− 0.078	7.84	**0.005**
AR * RD	− 0.29	10.93	**0.001**	0.08	0.73	0.394	0.096	9.05	**0.003**
AR * HP	− 0.01	0.03	0.858	0.09	0.57	0.451	0.098	6.10	**0.014**
RD * HP	0.50	4.50	**0.034**	0.24	5.13	**0.024**	0.066	4.16	**0.041**
Annual sample size	0.02	8.84	**0.003**	0.02	2.91	0.088	0.002	0.93	0.334

Mean number of alleles/locus and the four climatic variables were standardized within pool. Significant effects are indicated in bold.

The number of alleles per locus and observed heterozygosity were significantly affected by all climatic variables through their interactions (Table 1). Both diversity measures decreased with the increase in the annual number of rainy days during the shortest hydroperiod. During the longest hydroperiod the observed heterozygosity increased with the increase in the annual number of rainy days, but the number of alleles per locus was not affected. Observed heterozygosity also showed a decrease with the increase in annual rainfall, and an increase with the increase in mean January temperature, during the shortest hydroperiod. In contrast, during the longest hydroperiod, the observed heterozygosity showed an increase with the increase in annual rainfall, and a decrease with the increase in mean January temperature (Table 1).

### Annual variation in allele composition

Overall, alleles frequencies differed significantly among years in every pool we examined (CQ: ${\upchi }_{{122}}^{2}$ = 152.7, *P* = 0.028; GB: ${\upchi }_{{234}}^{2}$ = 415.4, *P* < 0.001; GC: ${\upchi }_{{219}}^{2}$ = 629.9, *P* < 0.001; GN: ${\upchi }_{{168}}^{2}$ = 262.8, *P* < 0.001; GO: ${\upchi }_{{106}}^{2}$ = 139.1, *P* = 0.012; GQ: ${\upchi }_{{208}}^{2}$ = 363.2, *P* < 0.001; LD: ${\upchi }_{{53}}^{2}$ = 120.4, *P* < 0.001; Fig. 2c). The results of the correspondence analysis (Fig. 2c) indicate that within the same pool, certain alleles were common in specific years and rare in other years. An illustration of this is presented in Fig. 2b, which demonstrates a sizable change among years in the frequency of four focal loci. For example, the frequency of allele G319 in the GO pool was 0.50 in 2013 and 0.24 in 2015. Alleles G323 and G327 in the same pool showed a similar magnitude of change between years, whereas alleles G307 and G334 showed no change in frequency. A similar pattern of change in frequency between years can be observed in the other pools presented in Fig. 2b and throughout our dataset.

The D_A_ distance, which quantifies the number of allelic differences between years, corroborated the results obtained by the correspondence analysis. In every pool, we observed significant differences between years (Table 2). For example, in pool GQ the D_A_ distances among years all significantly differed from each other, whereas in pool CQ the only significant D_A_ value was between 2013 and 2015 (Table 2). For pool LD, the D_A_ distance (0.219) between 2013 and 2015 was also significantly greater than that expected by random (*P* < 0.0001).

**Table 2:** Corrected Nei’s average number of differences in allele frequency between years within each pool (D_A_; above diagonal), and the associated P value (below diagonal).

Year	2010	2012	2013	2015		2007	2012	2013	2015
*GB*					*GC*				
2010	–	0.357	0.526	0.185	2007	–	0.182	0.456	0.133
2012	**0.003**	–	0.402	0.085	2012	0.125	–	0.341	0.136
2013	**0.001**	**< 0.001**	–	0.332	2013	**0.003**	**< 0.001**	–	0.374
2015	**0.018**	**0.014**	**< 0.001**	–	2015	0.137	**0.020**	**< 0.001**	–
*CQ*					*GO*				
2012		–	0.059	− 0.039	2012		–	0.088	0.116
2013		0.096	–	0.107	2013		0.199	–	0.084
2015		0.784	**0.005**	–	2015		**0.043**	0.066	–
*GN*					*GQ*				
2012		–	− 0.076	0.086	2012		–	0.218	0.158
2013		0.835	–	0.141	2013		**0.002**	–	0.093
2015		0.174	**0.007**	–	2015		**< 0.001**	**0.013**	–

Significant D_A_ distances are indicated in bold. Pool name abbreviations were taken from Table S2.

An overall locus-by-locus AMOVA revealed a significant temporal within pool component among years (2.5%, *P* < 0.0001). The spatial component (i.e. among pools) accounted for 12% of the total genetic variation (*P* < 0.0001), and an additional 2.3% (*P* < 0.0001) accounted for the genetic variance among individuals within years. The remaining unexplained variation occurred within individuals (Table S5).

Similar to diversity, we used the GEE mixed model approach to test for climatic effects on interannual allele frequency variation. Overall, we found annual rainfall and rainy days to be the sole significant variables affecting the annual change in allele frequency (Table 3). Surprisingly, none of the interactions we examined were significant. We repeated the above analysis separately for each locus, as selection may operate in a differential manner on different loci. We found that for loci Psy3, Psy 5, and Psy 17, the climatic variables did not significantly explain any of the interannual variation (Table S6). For the five other loci (Psy1, Psy2, Psy4, Psy6, Psy8) annual rainfall and rainy days were key climatic variables affecting interannual allele frequency variation either directly or through interactions. We found a significant effect of maximum January temperature only in loci Psy2 and Psy4, and a significant effect of hydroperiod only in loci Psy2 and Psy6 (Table S6).

**Table 3:** The effects of maximum January temperature (C), annual rainfall (mm), number of rainy days (> 0.1 mm/day), and length of hydroperiod (days) on the frequency of alleles.

Effects	Estimate	Wald ${\upchi }_{1}^{2}$	P	VI	VIF
Maximum January temperature (MJT)	0.094	0.456	0.500	0.13	3.6
Annual rainfall (AR)	0.183	16.643	**< 0.001**	0.41	1.2
Annual rain days (RD)	0.216	25.309	**< 0.001**	0.36	3.0
Hydroperiod (HP)	− 0.009	0.006	0.938	0.12	2.0
MJT * AR	0.026	0.017	0.897		
MJT * RD	− 0.262	1.922	0.166		
MJT * HP	0.031	0.068	0.794		
AR * RD	0.166	0.676	0.411		
AR * HP	0.050	0.121	0.728		
RD * HP	− 0.009	0.004	0.952		

The four climatic variables were standardized within each pool. Significant effects are indicated in bold. Variable importance is denoted by VI and the variance inflation factor by VIF.

## Discussion

Our hypothesis posited that the frequencies of tadpole genotypes would change according to the selective climatic pressure every year; and, therefore, we expected to see changes in allele frequencies among years. Indeed, both the correspondence analysis and the D_A_ distances revealed a significant difference in allele composition among years in all seven vernal pools (Fig. 2b,c, Table 2). Using mixed models, we have demonstrated that annual climatic variations partially account for the variation in allelic composition. Thus, climatic variables, either directly or via interaction among the predictors, affected the frequency of alleles in the vernal pools. Furthermore, we have also demonstrated that the number of alleles, evenness, and heterozygosity also varied between years in accordance to changes in the annual climate. These findings are far from trivial, because the number of alleles, evenness, and heterozygosity can also remain constant from year to year, while only the allele frequencies vary. However, our results suggest that selection by annual changes in climate is altering both the allelic composition and the shape of the distribution of allele frequency in each pool, as expected in a region with high climatic instability between years (Fig. 1a). Support for a selection process also comes from the fact that number of alleles in a pool significantly decreases with tadpole age, most likely due to the death of most tadpoles throughout the hydroperiod (Table S7, Fig. S1).

There are relatively few studies that have examined short-term genetic changes in a population (e.g.^38,39^). A recent study found temporal genetic instability between two consecutive years in an edge population of the western toad (*Anaxyrus boreas*^16^), the temporal component explained 2.4% of genetic variability, similar to the finding in the present study (2.5%; Table S5). The authors suggested that their results were an outcome of low philopatry in the western toad and the considerable turnover in pond use between years. The spadefoot toad, in contrast, is highly philopatric, with a dispersal ability of about 1 km for an individual^19,40^. Considering the spatial distribution of vernal pools and the dispersal ability of juveniles, most of the vernal pools present in Israel are assumed to house isolated populations^41^, as can also be seen from the low migration rates we found (Table S8). The temporal genetic variation we found is thus unlikely to have been caused by adult dispersal between breeding sites.

Savage et al.^13^ studied the southern long-toed salamander (*Ambystoma macrodactylum sigillatum*) in the Lake Tahoe basin, and found a significant genetic differentiation between successive years. Since the harsh landscape in that area restricts gene flow, the authors had expected that the small local breeding populations at each site would be relatively inbred and genetically consistent from year to year. They attributed their unexpected findings to high asymmetry in reproductive success among adults in a breeding deme; however, they did not offer an explanation for this variation in reproductive success. Waples and Teel^42^ showed that annual genetic variation in a population of Pacific salmon (*Oncorhynchus tshauytscha*), a highly philopatric breeder, fluctuated considerably (up to 36%) in a hatchery. They attributed this result to the relatively few adults which succeed in breeding every year (low effective population size), even though large number of adult salmons reached the hatchery. Similar pattern was shown by Chen et al.^43^ using a detailed long term (23 years) pedigree of Florida Scrub-Jays (*Aphelocoma coerulescens*) and SNPs data. They showed that annual allele frequency variations can be explained by change in survival and reproductive success of breeders, and that the contribution of gene flow is small. In the current study, however, if annual variation in allelic frequency was only associated with annual reproductive skew, we would have expected a lack of correlation with annual climate. One alternative explanation to our results is that annual climate conditions affect the breeding behavior of adult toads. Spadefoot toads reach sexual maturity only after 3–4 years^44^, and can live for 12 years in the wild^45^. Like many anuran species occupying dry habitats, breeding of *P. syriacus* is explosive, and takes place in temporary pools of water left by rainfall^19^. The adults synchronize the emergence from aestivation using the low frequency sound of rain hitting the ground^46^. Thus, the activity of breeding in spadefoot toads is limited to a few rainy nights^19^, giving no room for misopportunity, and most likely pushes the philopatric adults living around a pool to participate in breeding every year. Thus, it is less likely that the variation we observed in annual allelic frequency is due to reproductive skew.

Let us generalize even further. Our hypothesis posited that annual climatic variation would affect allele frequency. Extending this idea could suggest that populations living in regions of higher annual climate fluctuations would be expected to show greater annual variation in allele composition and diversity in their progeny; whereas, in contrast, populations in regions where annual fluctuations in climate are low, would be expected to show low annual variation in allele composition and diversity. One clear exception to this trend is the result of a catastrophic climatic event that can wipe out genetic variation altogether, but such extreme events are usually rare. The extent of annual fluctuations in climate is not evenly or randomly distributed across the globe. Examination of the map of annual fluctuation in rainfall on a global scale (Fig. 1a,^23^) reveals that certain areas on the globe show a high annual instability in rainfall. The largest area of rainfall instability on earth is the Saharo-Sind climatic zone (North Africa to the Indian desert; 60–160%). Other extensive regions of annual instability in rainfall (60–100%) are the Australian deserts, Namib in Africa, south-western North America, the Atacama Desert in South America, and the Mongolian Plateau (Fig. 1a). Other regions, including much of the tropics and the temperate zones, experience much less variation in annual rainfall between years. Israel is located at the northern edge of the Saharo-Sind climatic zone, and thus subjected to considerable interannual fluctuations in the amount of rainfall, the number of rainy days, and the extent of the hydroperiod. In other words, the location of our study site is a key factor. Thus, carrying out a similar study to ours in the temperate zone of Europe or North America might not reveal strong correlations between annual climatic variables and annual fluctuations in genetic diversity and composition, because the interannual variation in climate in the temperate zone is small (20–30%).

Last, the results of our and previous studies highlight the importance of temporal sampling in population genetics research. Although the temporal variance component accounts for only about 2% of the total genetic variance, it accounts for about 15% of the total explained genetic variance (Table S5). Thus, the climate fluctuations could account for a considerable percentage of the genetic variance we can explain. Surprisingly, the effect of short-term climate fluctuations on genetic composition have sparsely been addressed in the literature, and have been underrated. Our results suggest that drawing genetic conclusions regarding a population based on a single year of sampling can be flawed, especially for studies conducted in zones subjected to unstable climatic regimes. For example, strong isolation by distance was detected in 2006 in an African cichlid fish population in Uganda^47^, but was not evident in a follow-up study in 2008^48^. Repeated sampling in successive years is especially important when sampling the larvae of species in which not every adult breeds every year^13,16,49^, or when populations are exposed to strong seasonal or interannual variation in abiotic conditions^12,48^. The temporal genetic variation could become emphasized in small isolated populations due to increased effects of genetic drift and inbreeding^12^. In the present study, private alleles were identified in most of the years (Table S9). Private alleles are rare alleles that were found in a particular pool in only one year during the study period. Sampling during a single year could result in a lack of a true representation of the genetic variability of a pool, because the full range of alleles for a *P. syriacus* tadpole population in a given pool is not available in any single year. Thus estimates based on samples taken at one point in time might not be valid across time, whereas sampling over several successive years may lead to more accurate estimates of a population's genetic structure.

## Supplementary Information

Supplementary Information.

Supplementary Information.

## Data Availability

All genetic data used are available as a supplementary table.
