# Supplementary material for: Annual climatic fluctuations and short-term genetic variation in the eastern spadefoot toad
Source: Sci Rep. 2021 Jun 29;11:13514. doi: 10.1038/s41598-021-92696-w (PMC8241830; doi:10.1038/s41598-021-92696-w)
Supplement: Supplementary file 1 — Supplementary Information. [file 41598_2021_92696_MOESM1_ESM.pdf]

Annual climatic fluctuations and short-term genetic variation in the eastern spadefoot toad.

Orly Cohen[1], Yoav Ram[1], Lilach Hadany[2], Sarig Gafny[3§], Eli Geffen[1§]

[1]*School of Zoology, Tel Aviv University, Tel Aviv 69978, Israel*

[2]*School of Plant Sciences and Food Security, Tel Aviv University, Tel Aviv 69978. Israel*

[3]*Faculty of Marine Sciences, Ruppin Academic Center, Michmoret 40297, Israel*

[§] equal contribution

Table S1: Characterization of eight new polymorphic microsatellite loci in the eastern spadefoot toad (*P. syriacus*). Primer sequences, size of amplified product, number of individuals successfully genotyped (N), number of alleles (Na), observed (Ho) and expected (He) heterozygosity.

Locus	Primer sequence (5'-3')	Size range (bp)	N	Na	Ho	He
Psy1	F: CGCTCATTCACTAAACAGCAATTTC<br>R:TTGACCTTGGCATTTAGAGAAGAAG	138-227	773	20	0.789	0.699
Psy2	F: TTCATCTATTCATTCCTCCATGTCC<br>R:TCTTTCTCATACTGACCACATTGATG	134-288	757	36	0.475	0.755
Psy3	F: TTACAAATAGGCCACATAGTTGCTG<br>R: TTGCTCTTCTTCCAGATTAATGTTC	156-266	772	20	0.809	0.747
Psy4	F: CATCTGTAATACGACTTCCCATTGG<br>R:GAACAGCACCTCCATATAGACAGAC	190-312	773	30	0.837	0.762
Psy5	F:TGAAATAAACCTACAGTGCAACATTTC<br>R:TTCAGGGAATATGAGAAAGGCATTG	223-316	773	19	0.603	0.590
Psy6	F: TTTACCTGCGTGTGTATTATACCTG<br>R: TTATGAATGTTCTCAGATGTGGGTG	295-384	767	21	0.806	0.765
Psy8	F: TCTGTGACTCCTACTTCAACATCAG<br>R:AGATGCTGTGTAGTCAATGTGAATG	292-360	768	28	0.786	0.737
Psy17	F:CTAGCATATATCTCTAGGTCACACTG<br>R:CCCAACACCAAGTATTTCTAAGTATTG	204-283	770	19	0.807	0.761

We looked for loci with tetramer motifs, range size of 150-400bp, annealing temperature of 58-60ºC, and 7-10 alleles. Ten primers were initially chosen for the next step of PCR testing. The primers were tested in a 25ul PCR mix containing 12.5ul KAPA2G Fast ReadyMix (KAPA Biosystems), 10uM of forward and reverse primers, 1ul of DNA and 9ul of PCR-grade water. The PCR cycling protocol included an initial denaturation step (3 min, 95ºC), followed by 35 cycles of denaturation (15 sec, 95ºC), annealing (15 sec, 57ºC), and extension (15 sec, 72ºC), and a final extension step (3 min, 72ºC). After preliminary testing, we retained eight optimal primers (Table S1). Forward primers were labeled with fluorescent dyes (6-FAM, Tamra, HEX, and ROX) for use in two multiplex reactions. Genotyping was performed using an ABI 3500xl Genetic Analyzer with the GeneScanT 600 LIZ size standard (Applied Biosystems). Peaks were scored manually in GeneMarker (version 1.97, SoftGenetics).

Table S2: Vernal pools, locations, elevation (m above sea level), and IMS meteorological stations. Climate data were taken from the closest IMS meteorological station to each vernal pool (MJT, AR and RD columns). In cases where there was not a single proximate station to the pool, we used an average of the records from two or three IMS stations in the area.

Vernal pool	Latitude	Longitude	elevation	MJT	AR and RD
Robert's (CQ)	31.752824	34.649076	30	Nitzan	Nitzan, Nitzanim
Golani (LD)	32.781054	35.407351	220	Deir Hana	Lavi
Khushniya (GO)	32.996878	35.810829	750	Merom Golan	Keshet
Rasaniya (GQ)	33.026706	35.756558	663	Merom Golan	Keshet
Surman down (GN)	33.099252	35.834856	970	Merom Golan	Alonei Habashan
El Rom (GC)	33.175410	35.775974	1040	Merom Golan	El Rom
Zaura (GB)	33.222525	35.720992	780	Merom Golan	Banias

MJT= maximum January temperature; AR= annual rain; RD= number of rainy days (>0.1 mm).

Table S3: Correlation (Spearman r_s) tests between the four environmental variables. Significant P-values are indicated in bold.

Variable 1	Variable 2	N	r_s	P
HP	RD	20	0.465	**0.039**
RD	AR	20	0.404	0.077
HP	AR	20	0.288	0.218
HP	MJT	20	-0.186	0.433
AR	MJT	20	-0.307	0.189
RD	MJT	20	-0.707	**<0.001**

MJT= maximum January temperature; AR= annual rain; RD= number of rainy days (>0.1 mm/day); HP= length of hydroperiod (days).

Table S4: List of the names (abbreviation) of seven vernal pools sampled in this study. For each site, we calculated the average number of alleles per locus (Na), the Shannon's information evenness (J), and the observed heterozygosity (Ho). In parenthesis is the number of samples per year.

Pool	Na			J			Ho		
Year	2012	2013	2015	2012	2013	2015	2012	2013	2015
Zaura (GB)	8.3 (17)	5.8 (16)	9.1 (51)	1.71	1.42	1.71	0.63	0.69	0.73
El Rom (GC)	5.9 (13)	6.0 (30)	8.8 (57)	1.54	1.47	1.77	0.80	0.75	0.76
Surman down (GN)	6.1 (6)	7.4 (15)	9.1 (39)	1.66	1.72	1.87	0.83	0.75	0.79
Rasaniya (GQ)	7.3 (15)	6.6 (15)	12.5 (67)	1.62	1.55	1.99	0.77	0.75	0.75
Khushniya (GO)	4.1 (11)	5.5 (16)	5.8 (36)	1.13	1.33	1.26	0.63	0.55	0.63
Golani (LD)	6.0 (30)	5.9 (30)		1.46	1.42		0.73	0.78	
Robert's (CQ)	6.3 (15)	7.4 (41)	6.5 (20)	1.60	1.62	1.62	0.74	0.78	0.80

Table S5: AMOVA results for temporal and spatial variance. The variance component (VC) and percentage of each source of variation out of the total and explained variance.

Source of variation	VC	% of total variation	% of explained variation	P
Among pools	0.43	12.06	71.27	<0.0001
Among years within pool	0.09	2.49	14.72	<0.0001
Among tadpoles within year	0.08	2.32	13.71	<0.0001
Within (i.e. unexplained)	2.95	83.12		<0.0001
Total	3.54			

Table S6: The effects of maximum January temperature, annual rainfall, number of rainy days (>0.1 mm/day), and length of hydroperiod (days) on the frequency of alleles by locus. The four climatic variables were standardized within each pool. Significant effects are indicated in bold.

Effects	Estimate	Wald χ^2_1	P
Psy1			
Maximum January temperature (MJT)	0.116	0.185	0.667
Annual rainfall (AR)	0.170	4.788	**0.029**
Annual rainy days (RD)	0.163	1.373	0.241
Hydroperiod (HP)	-0.031	0.023	0.879
MJT * AR	-0.001	0.000	0.998
MJT * RD	-0.263	0.936	0.333
MJT * HP	-0.038	0.052	0.820
AR * RD	0.176	0.602	0.438
AR * HP	-0.036	0.031	0.860
RD * HP	-0.077	0.178	0.673
Psy2			
Maximum January temperature (MJT)	0.552	16.091	**<0.001**
Annual rainfall (AR)	0.150	18.188	**<0.001**
Annual rainy days (RD)	0.344	8.431	**0.004**
Hydroperiod (HP)	-0.208	1.780	0.182
MJT * AR	-0.452	9.449	**0.002**
MJT * RD	0.053	0.047	0.828
MJT * HP	0.373	5.572	**0.018**
AR * RD	-0.310	2.018	0.155
AR * HP	0.256	4.404	**0.036**
RD * HP	-0.007	0.002	0.967
Psy3			
Maximum January temperature (MJT)	-0.215	1.138	0.286
Annual rainfall (AR)	0.089	2.120	0.145
Annual rainy days (RD)	0.057	0.406	0.524
Hydroperiod (HP)	0.271	1.852	0.174
MJT * AR	0.098	0.354	0.552
MJT * RD	-0.594	2.193	0.139
MJT * HP	0.072	0.213	0.644
AR * RD	0.191	0.959	0.327
AR * HP	0.094	0.591	0.442
RD * HP	0.105	0.103	0.748
Psy4			
Maximum January temperature (MJT)	-0.341	4.297	**0.038**
Annual rainfall (AR)	0.337	22.124	**<0.001**
Annual rainy days (RD)	0.196	7.120	**0.008**
Hydroperiod (HP)	0.116	1.510	0.219
MJT * AR	0.482	3.722	0.054
MJT * RD	-0.503	10.708	**0.001**
MJT * HP	-0.326	3.587	0.058
AR * RD	0.546	5.095	**0.024**
AR * HP	-0.244	1.655	0.198
RD * HP	0.072	0.247	0.619

Psy5			
Maximum January temperature (MJT)	0.086	0.204	0.651
Annual rainfall (AR)	0.138	1.241	0.265
Annual rainy days (RD)	0.114	0.941	0.332
Hydroperiod (HP)	-0.077	0.166	0.684
MJT * AR	0.149	0.248	0.619
MJT * RD	-0.213	0.506	0.477
MJT * HP	0.172	0.974	0.324
AR * RD	0.199	0.473	0.492
AR * HP	-0.216	1.018	0.313
RD * HP	0.222	1.046	0.306
Psy6			
Maximum January temperature (MJT)	0.108	0.185	0.667
Annual rainfall (AR)	0.236	69.078	**<0.001**
Annual rainy days (RD)	0.332	14.268	**<0.001**
Hydroperiod (HP)	-0.005	0.001	0.981
MJT * AR	0.003	0.000	0.986
MJT * RD	-0.334	1.184	0.276
MJT * HP	-0.099	0.340	0.560
AR * RD	0.190	1.097	0.295
AR * HP	0.275	3.917	**0.048**
RD * HP	-0.054	0.032	0.858
Psy8			
Maximum January temperature (MJT)	0.347	3.377	0.066
Annual rainfall (AR)	0.183	11.376	**0.001**
Annual rainy days (RD)	0.366	26.401	**<0.001**
Hydroperiod (HP)	-0.136	0.899	0.343
MJT * AR	-0.048	0.025	0.874
MJT * RD	-0.240	1.185	0.276
MJT * HP	0.061	0.083	0.773
AR * RD	0.261	0.812	0.367
AR * HP	0.032	0.023	0.878
RD * HP	-0.151	0.577	0.448
Psy17			
Maximum January temperature (MJT)	0.121	0.644	0.422
Annual rainfall (AR)	0.110	2.061	0.151
Annual rainy days (RD)	0.088	1.183	0.277
Hydroperiod (HP)	-0.031	0.134	0.714
MJT * AR	0.032	0.021	0.885
MJT * RD	0.039	0.064	0.800
MJT * HP	0.117	0.682	0.409
AR * RD	0.113	0.380	0.538
AR * HP	0.078	0.312	0.577
RD * HP	-0.100	0.548	0.459

Table S7: The effect of tadpole age and pool name on standardized number of alleles, Shannon's evenness, and observed heterozygosity (Ho). P-values were calculated by randomizations. Variable importance was evaluated using total effect.

Term	*df*	F	P	Total effect
Standardized number of alleles				
Pool	6,7	3.9	**0.038**	0.72
Tadpole age	1,13	71.8	**<0.001**	0.67
Pool * Tadpole age	6,7	18.6	**<0.001**	
Shannon's evenness				
Pool	6,7	2.2	0.178	0.96
Tadpole age	1,13	0.3	0.604	0.36
Pool * Tadpole age	6,7	1.3	0.374	
Observed heterozygosity				
Pool	6,7	3.2	0.101	1.00
Tadpole age	1,13	0.0	0.999	0.23
Pool * Tadpole age	6,7	0.9	0.557	

Table S8: Migration rates among the studied vernal pools in northern Israel. The percentage of individuals in each pool (rows) that are migrants from a source pool (columns). Recent migration rates among pools were estimated using the software BayesAss (version 3.0; Wilson and Rannala 2003).

Pool	GB	GC	GN	GO	GQ	LD
GB	**95.10**	0.44	0.34	0.39	0.51	0.43
GC	0.45	**95.00**	0.47	0.28	0.99	0.26
GN	0.89	1.46	**91.33**	0.58	1.08	0.41
GO	0.43	0.46	0.47	**93.12**	1.27	0.46
GQ	0.47	0.47	1.17	0.61	**94.1**1	0.34
LD	0.40	0.43	0.45	0.44	0.46	**93.85**

Wilson, G. A., Rannala, B. (2003) Bayesian inference of recent migration rates using multilocus genotypes. *Genetics 163*, 1177–1191.

Table S9: The number of private alleles sampled in each year, and number of loci demonstrating private alleles (NL_{PA}). Private alleles (PA) are those that appeared in a pool only in a particular year. In this analysis each vernal pool is treated separately. N=sample size.

Pool	Year	N	PA	NL_{PA}
CQ	2012	15	0	0
	2013	41	6	5
	2015	20	3	2
GB	2010	6	0	0
	2012	17	3	3
	2013	16	1	1
	2015	51	7	5
GC	2007	5	1	1
	2012	13	1	1
	2013	30	0	0
	2015	57	7	4
GN	2012	6	6	3
	2013	15	4	3
	2015	39	17	7
GO	2012	11	1	1
	2013	16	6	5
	2015	36	7	4
GQ	2012	15	3	2
	2013	15	0	0
	2015	67	33	7
LD	2013	30	7	6
	2015	30	6	5

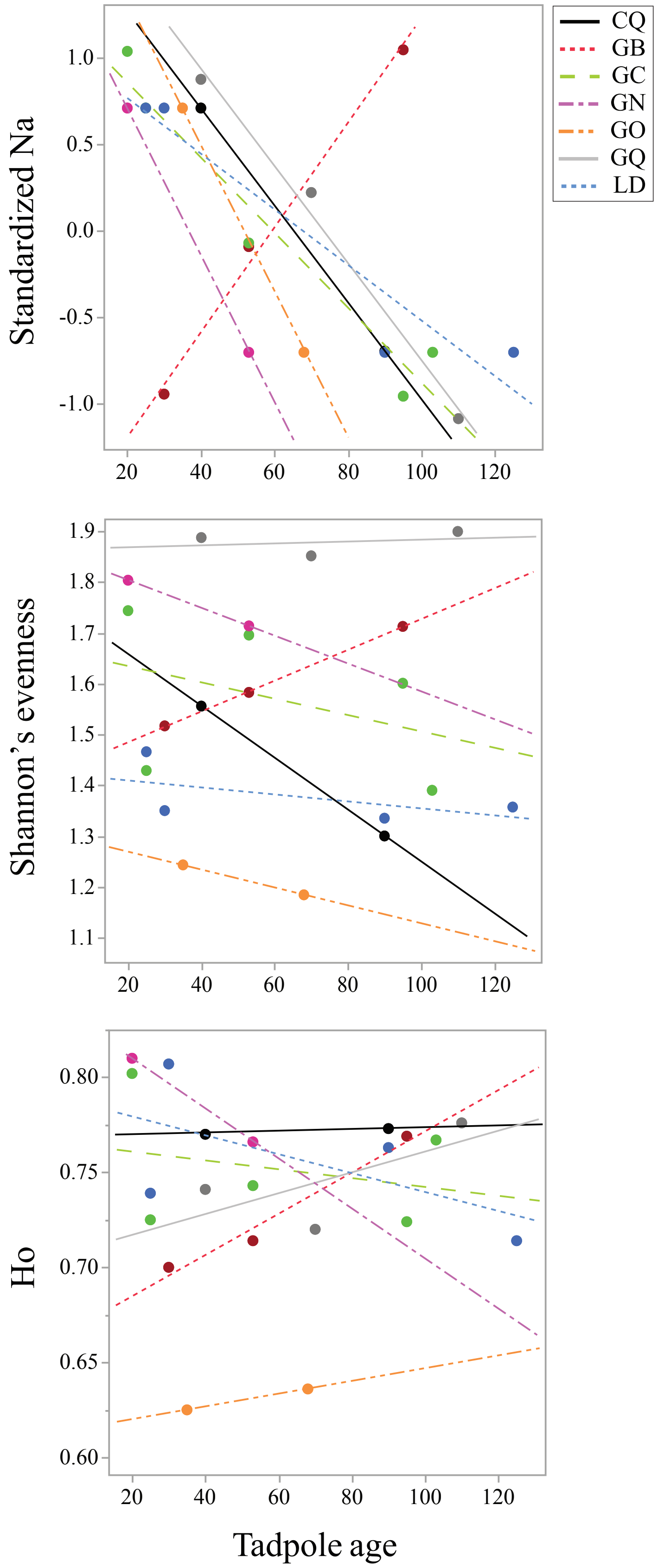

Fig. S1: The change in the standardized number of alleles, Shannon's evenness, and observed heterozygosity (Ho) as a function of tadpole age (days). Pool names and regression slopes are denoted by color and line type.
